# Identification of genetic variants associated with anterior cruciate ligament rupture and AKC standard coat color in the Labrador Retriever

**DOI:** 10.1186/s12863-023-01164-z

**Published:** 2023-10-26

**Authors:** BT Lee, LA Baker, M Momen, H Terhaar, EE Binversie, SJ Sample, Peter Muir

**Affiliations:** Department of Surgical Sciences, University of Wisconsin-Madison, School of Veterinary Medicine, 2015 Linden Drive, Madison, WI 53706 United States of America

**Keywords:** Genetics, Anterior cruciate ligament rupture, Genome-wide association study, Dog, Polygenic disease

## Abstract

Canine anterior cruciate ligament (ACL) rupture is a common complex disease. Prevalence of ACL rupture is breed dependent. In an epidemiological study, yellow coat color was associated with increased risk of ACL rupture in the Labrador Retriever. ACL rupture risk variants may be linked to coat color through genetic selection or through linkage with coat color genes. To investigate these associations, Labrador Retrievers were phenotyped as ACL rupture case or controls and for coat color and were single nucleotide polymorphism (SNP) genotyped. After filtering, ~ 697 K SNPs were analyzed using GEMMA and mvBIMBAM for multivariate association. Functional annotation clustering analysis with DAVID was performed on candidate genes. A large 8 Mb region on chromosome 5 that included *ACSF3*, as well as 32 additional SNPs, met genome-wide significance at *P* < 6.07E-7 or Log_10_(BF) = 3.0 for GEMMA and mvBIMBAM, respectively. On chromosome 23, SNPs were located within or near *PCCB* and *MSL2*. On chromosome 30, a SNP was located within *IGDCC3*. SNPs associated with coat color were also located within *ADAM9*, *FAM109B*, *SULT1C4*, *RTDR1*, *BCR*, and *RGS7*. *DZIP1L* was associated with ACL rupture. Several significant SNPs on chromosomes 2, 3, 7, 24, and 26 were located within uncharacterized regions or long non-coding RNA sequences. This study validates associations with the previous ACL rupture candidate genes *ACSF3* and *DZIP1L* and identifies novel candidate genes. These variants could act as targets for treatment or as factors in disease prediction modeling. The study highlighted the importance of regulatory SNPs in the disease, as several significant SNPs were located within non-coding regions.

**Supplementary Information:**

The online version contains supplementary material available at 10.1186/s12863-023-01164-z.

## Introduction

The cruciate ligaments within the knee provide stability and oppose rotational and translational forces. In humans, the anterior cruciate ligament (ACL) is prone to rupture mostly through non-contact injury, especially in young female athletes [1]. In dogs, the cranial cruciate ligament is anatomically equivalent to the ACL and is also vulnerable to non-contact injury [2]. ACL rupture is the most common cause for canine lameness [3]. The underlying disease mechanism for most cases is described as the progressive tearing of ligament fibers in the presence of stifle synovitis [4, 5]. A combination of genetic and environmental factors defines an individual’s risk of complex disease development. Established genetic risk factors include breed, joint conformation, and joint immune responses, while age, obesity, and other extrinsic factors also have roles in disease development [6–9]. Prevalence is breed dependent; ACL rupture is more common in large breed dogs, such as Newfoundlands, Rottweilers, and Labrador Retrievers [9]. Heritability estimates for canine ACL rupture range from 0.27 to 0.89 [10–13]. High prevalence in a breed is a consequence of intense selection leading to a concentration of risk loci [14]. Linkage disequilibrium (LD) is extensive in dogs, which enhances the statistical power of genome-wide association studies (GWAS) using single nucleotide polymorphism (SNP) markers as an approach for variant discovery [15]. ACL rupture in the Labrador Retriever is a polygenic complex disease whereby many loci with small and moderate effects influence disease risk [12, 16]. Several candidate genetic variants have been identified [12, 17–19], but given its polygenic nature, it is likely that additional variants remain to be discovered.

Labrador Retrievers have three AKC recognized coat colors: black, chocolate, and yellow. Risk of ACL rupture is increased in yellow Labrador Retrievers compared to black and chocolate dogs [20]. Inheritance of coat color is controlled by two genes: *MC1R* and *TYRP1*. Yellow coat color is inherited in an autosomal recessive manner and is caused by a loss of function mutation in *MC1R* [21, 22]. In dogs that are homozygous for *MC1R* mutations, pheomelanin is produced, creating a yellow coat color, while dogs with at least one wildtype *MC1R* allele produce eumelanin resulting in a black or chocolate phenotype. In these dogs, color is determined by the *TYRP1* gene. Chocolate color is autosomal recessive to black and is seen with any of three *TYRP1* mutations [23]. Through LD, selection for coat color may have inadvertently selected risk variants for other phenotypes. For example, research suggests that chocolate Labrador Retrievers have shorter lifespans and are affected with skin and ear disease at higher rates than black or yellow Labrador Retrievers [24]. Behavioral differences have been associated with coat color as well [25]. Alternatively, genes that regulate coat color may have additional unknown direct biologic effects on ACL rupture risk. Studies have shown that *MC1R* has an important role in regulating inflammatory pathways [26–28], and variants associate with osteoarthritis [29].

High LD and diversity within and between dog breeds enhance use of GWAS as an approach for causal variant discovery. Joint analysis of multiple phenotypes is a valuable technique that can increase statistical power to detect small and moderate associations expected with complex traits, such as ACL rupture [18, 30], and detect associations with ACL rupture, coat color, or both phenotypes in this study. Such knowledge will advance analysis of candidate variants that could improve genomic prediction of disease and further mechanistic study of influential biological pathways.

## Materials and methods

### Research approach

A within-breed GWAS in the Labrador Retriever was performed by analyzing SNPs from case and control dogs. All procedures were performed in accordance with the recommendations in the Guide for the Care and Use of Laboratory Animals of the National Institutes of Health and the American Veterinary Medical Association and with approval from the Animal Care Committee of the University of Wisconsin-Madison (protocols V1070, V5463). Informed consent of each owner was obtained before participation in the study. Phenotypes included ACL rupture case/control status and coat color. Contact with the owners of control dogs was maintained to ensure accurate phenotyping and coding was updated if a dog became affected. Preparation of the manuscript conformed with the ARRIVE guidelines.

### Inclusion and exclusion criteria for phenotyping

Most Labrador Retrievers exhibit signs of ACL rupture by 8 years old [31]. Lameness, knee instability, and ligament damage are usually confirmed during surgical treatment. Dogs were excluded as a case if contact injury was diagnosed from the clinical history. Phenotype negative controls were dogs ≥ 8 years old with normal orthopaedic exams and normal knee radiographs.

Clinical examination of each dog was performed, including knee palpation for instability. Bilateral weight-bearing radiographs were reviewed and graded for stifle effusion and osteophytosis [32]. Dog age, sex, weight, neuter status, and coat color were also recorded [18].

### DNA isolation, SNP genotyping, and quality control

DNA was isolated from buffy coat leukocytes from EDTA blood or cheek swab saliva. DNA was isolated using standard reagents (Blood – Puregene Cell Core Kit, Qiagen, Germantown, MD; saliva – DNA Genotek prepIT-L2P, Ottawa, ON, Canada). Genotyping was performed using the Illumina Canine HD BeadChip which has ~ 220,000 SNPs mapped to CanFam3.1. SNPs were then imputed to the Thermofisher Axiom HD Canine 770 K array using Beagle 5.0 [19, 33] and a multi-breed reference panel that consisted of 646 purebred dogs of various breeds that were genotyped using the Axiom Canine HD 770 K array, including 96 Labrador Retrievers. Before imputation of the ACL rupture SNP data, we validated our method using a group of 22 Labrador Retrievers with whole genome sequence (WGS) data. Illumina SNPs were extracted from WGS data and imputed to denser set of Axiom SNPs using Beagle 5.0 with the reference panel, a window size of 3 cM with 1 cM overlap, and effective population size of 100. Imputation accuracy was 96%. Using PLINK1.9 [34], SNPs with a minor allele frequency (MAF) < 0.005 (to analyze potential rare variants) and a call rate < 95%, and dogs with a call rate of < 95% were removed. SNPs with deviations from Hardy-Weinberg proportions at *P* < 1E-7 were also filtered out.

### Multivariate GWAS

ACL rupture case-control and coat color phenotypes were used for multivariate association using the linear mixed model approach implemented in GEMMA [35]. P-values are calculated to measure support for each model compared to the null (no association). The value for genome-wide significance was calculated using a Bonferroni correction for the number of haplotype blocks in the genome using PLINK [34] and was determined to be *P* < 6.07E-7. Additionally, a Bayesian statistical model was used with mvBIMBAM [36] to identify associations between genotypes and phenotypes (directly associated, indirectly associated, or unassociated with one or both phenotypes). Bayes Factors were evaluated for evidence of association with the multivariate phenotype. SNPs with a Log_10_ Bayes Factor > 3 were considered to have moderate evidence of association, and SNPs with a Log_10_ Bayes Factor > 6 were considered to have strong evidence of association. Marginal posterior probabilities of associated SNPs were evaluated to determine which phenotypes may be influencing the association. Sex, age, neuter status, and weight were included as covariates in both models. For mvBIMBAM, case-control phenotypes were residuals of multiple logistic regression against the covariates.

Regions with evidence of association with either GEMMA or mvBIMBAM analysis were evaluated using the CanFam3.1 Broad Improved Canine Annotation catalog in the UCSC Table Browser to identify associated genes. LocusZoom [37] and triangle heat map plotting with the ‘gpart’ R package and the Big-LD algorithm [38, 39] were also used to investigate haplotype structure and candidate genes in selected regions.

### Functional annotation clustering

A list of genes that were within a ±25 kb flanking region of each significant SNP was created using the canFam3.1 Broad Improved Canine Annotation catalog in the UCSC Table Browser. Functional annotation clustering was then performed using DAVID [40, 41]. Functional clusters with significant P-values were evaluated for biological relevance to the ACL rupture and coat color phenotypes.

## Results

Phenotype data were collected from 367 dogs. After quality control, the final dataset included 696,846 SNPs from 148 cases and 219 controls. There were 41 cases and 96 controls with a black coat color, 33 cases and 51 controls with a chocolate coat color, and 73 cases and 73 controls with a yellow coat color. Black dogs had a decreased risk of ACL rupture (odds ratio = 0.50, *P* = 0.0024), while yellow dogs had an increased risk (odds ratio = 1.99, *P* = 0.0017). Chocolate color did not influence ACL rupture risk (Table 1).

**Table 1:** Distribution of coat colors among ACL rupture case and control Labrador Retrievers

Coat Color	Cases	Controls	Odds Ratio	*P*-value
**Black**	41 (29.9%)	96 (70.1%)	0.50	< 0.005
**Chocolate**	33 (39.3%)	51 (60.7%)	0.96	0.87
**Yellow**	73 (50.0%)	73 (50.0%)	1.99	< 0.005

**Fig. 1:**
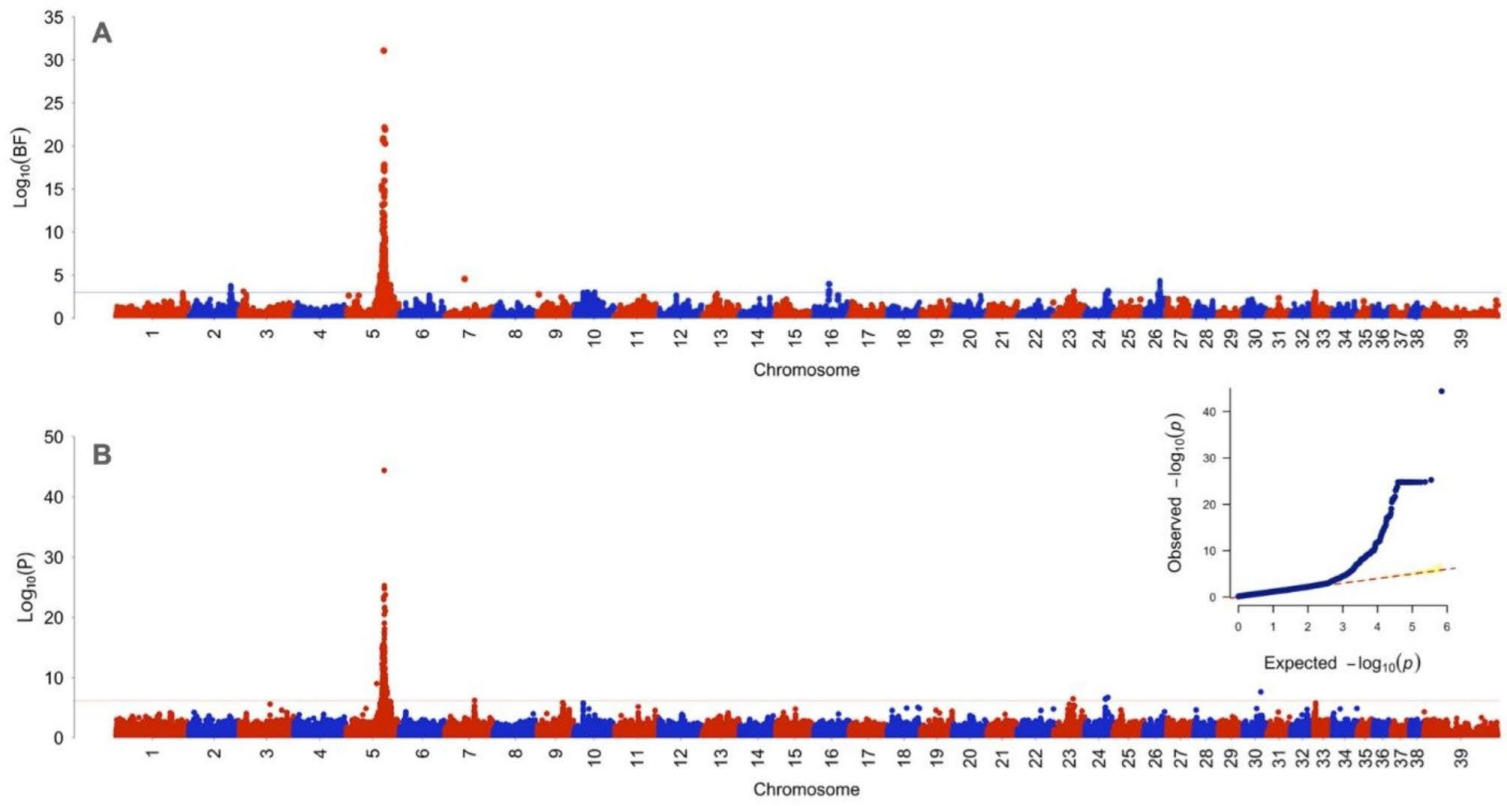
Manhattan plot of mvBIMBAM and GEMMA results for the multivariate phenotype of anterior cruciate ligament rupture and coat color. (**A**) Data for the mvBIMBAM analysis is shown as log_10_Bayes factors (BF) and (**B**) GEMMA data are displayed as log_10_P with the associated QQ plot of expected and observed *P*-values for GEMMA analysis to assess population stratification. SNPs on chromosome 5 displayed the most significant associations with the multivariate phenotype. There were also SNPs on chromosomes 2, 3, 7, 10, 16, 23, 24, 26, and 30 that showed significant association. Genome-wide significance cut-off is shown at Log_10_(BF) = 3.0 and -Log_10_(P) = 6.22. Lamda = 2.033

**Fig. 2:**
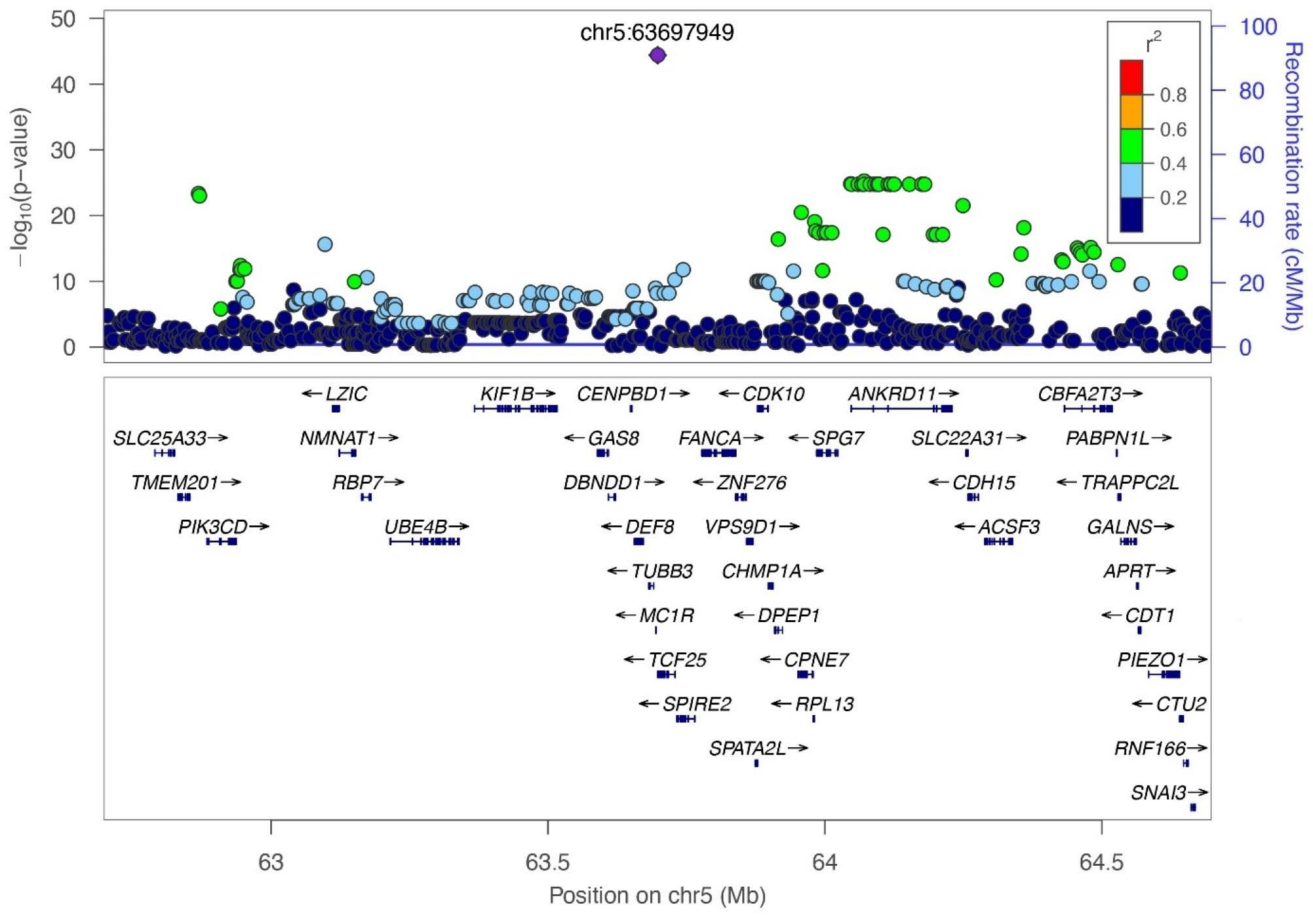
LocusZoom plot of the chromosome 5 candidate region illustrating linkage disequilibrium around *MC1R*. An ~ 3 Mb region centered around the most significant SNP is highlighted in this plot. SNPs with the highest LD were within the *ANKRD11*, *SPG7*, *CBFA2T3*, *ACSF3*, and *TMEM201* genes

**Fig. 3:**
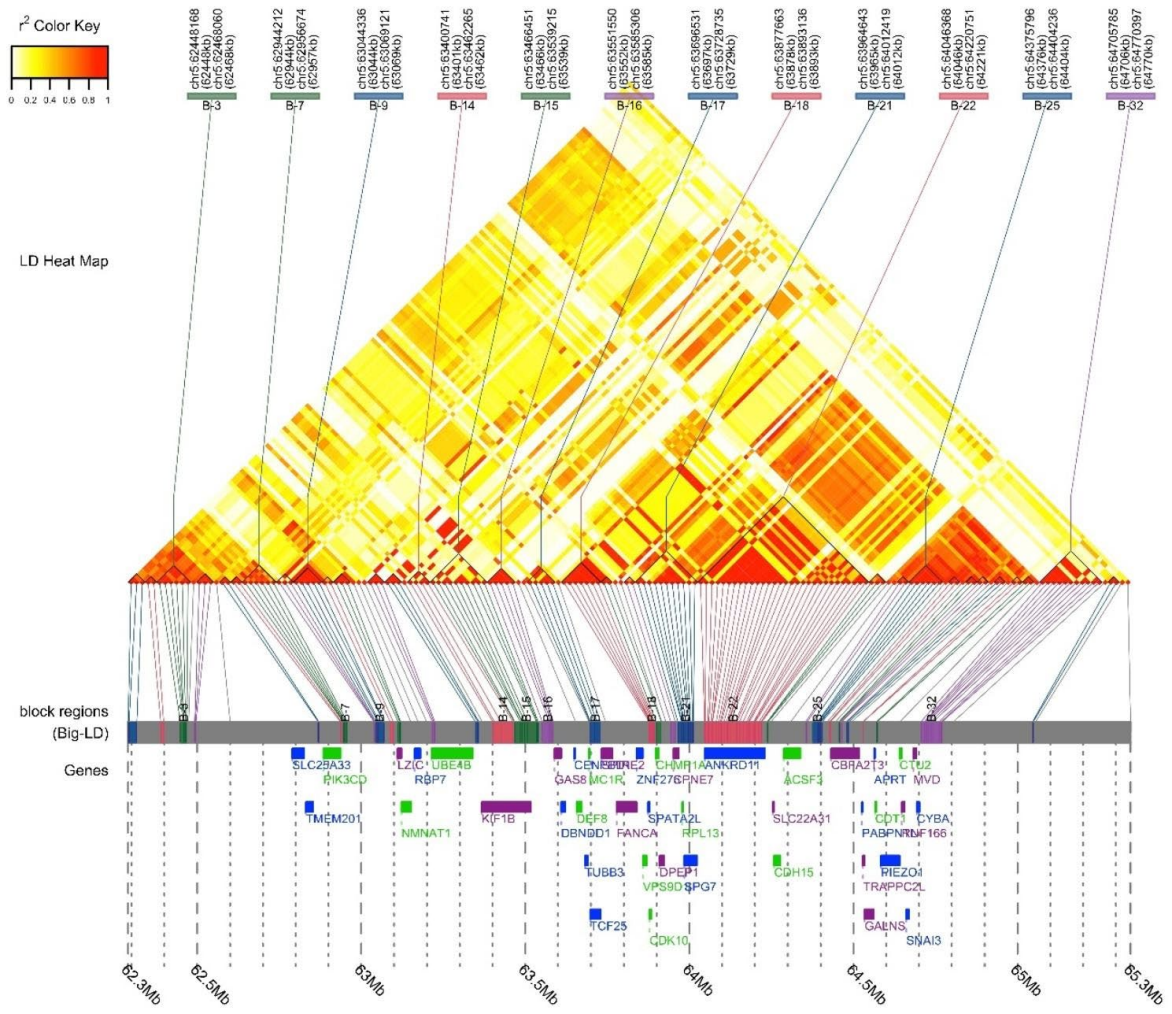
Triangle plot displaying haplotype blocks and linkage disequilibrium (LD) in the chromosome 5 candidate region. Regions with strong LD denoted by red coloring have the strongest associations and lighter colors have weaker LD. Haplotype blocks are outlined by triangular blocks. The *MCIR* and *TCF25* genes reside in the same haplotype block from chr5:63,694,334–63,728,735

The multivariate GWAS using GEMMA provided evidence of association (*P* < 6.02E-7) for 337 SNPs (Figs. 1 and 2; Table 2, Supplementary File S1). Of these, 331 were located on chromosome 5, including the most significant SNP (*P* = 3.95E-45), located within the *TCF25* gene, which is a transcription factor that is important in embryonic development. Significant SNPs on chromosome 5 spanned a large region of > 8 Mb that included more than 100 genes. The 6 other significant SNPs were located on chromosomes 7, 23, 24, and 30. SNPs were within or near non-coding or uncharacterized regions on chromosome 7 and 24 (Table 2). On chromosome 23, the association was near the *PCCB* and *MSL2* genes. On chromosome 30, the association was within the *IGDCC3* gene. To better discern nearby genes and haplotype structure in the large region chromosome 5, a LocusZoom plot was built around the most significant SNP (*P* = 3.95E-45) at chr5:63697949 (Fig. 3). There were many SNPs in LD within the *ANKRD11* gene. There were also SNPs in LD within *MC1R*, *PIK3CD*, *DPEP1*, *SPG7*, *ACSF3*, and *CBFA2T3* (Fig. 3). Additionally, a triangle plot was built spanning a ~ 3 Mb region (62.3-65.3 Mb) which revealed several areas of LD, including a haplotype block that contained *TCF25* and *MC1R*.

**Table 2:** SNPs associated with the multivariate phenotype of ACL rupture and coat color in the Labrador Retriever using linear mixed model GWAS with GEMMA [35]

Chr	Position	Number of SNPs	P-value	Gene	Location	Distance (bp)
5	63,697,949	1	3.95E-45	*TCF25*	63,697,627–63,729,780	0
5	64,046,368–64,095,007	9	5.48E-26–1.64E-25	*ANKRD11*	64,047,188–64,229,582	0
7	49,455,960	1	5.97E-07	lncRNA	49,407,711–49,493,131	0
23	32,670,460	1	3.19E-07	*PCCB*	32,689,873–32,782,007	19,413
				*MSL2*	32,631,936–32,662,611	7849
24	34,877,398	1	3.14E-07	non-coding	n/a	n/a
24	34,983,528	1	3.62E-07	non-coding	n/a	n/a
24	38,868,995	1	1.83E-07	non-coding	n/a	n/a
30	29,741,631	1	2.25E-08	*IGDCC3*	29,702,195–29,743,079	0

Note: There was a large area spanning approximately 8 Mb on chromosome 5 that contains 331 SNPs, including the one with the most significant association. Only the top 10 SNPs on chromosome 5 are presented in the table. Genes within ±25 kb of each SNP are reported

The multivariate analysis using mvBIMBAM revealed a total of 589 SNPs directly associated with either coat color, ACL rupture, or both (Fig. 1, Supplementary File S2). 237 of these SNPs displayed strong evidence of association (Log_10_ BF≥6.0). Of the associated SNPs, 556 were on chromosome 5, and were within or near 56 genes. The SNP with the highest significance (Log_10_ BF = 31.30) was located on chromosome 5 at the same position as the most significant SNP revealed in GEMMA analysis (Table 3) and was associated with coat color. There were nine additional loci associated with coat color found on chromosomes 2, 3, 7, 10, 16, and 26 (Table 3). The associations on chromosomes 2 and 3 were within noncoding regions. On chromosome 7, an association was identified within *RGS7*. Chromosome 10 associations were within *FAM109B* and *SULT1C4*. Three SNPs were associated *ADAM9* on chromosome 16. On chromosome 26, there were associations within lncRNA sequences, and the *RTDR1* and *BCR* genes. We identified one *DZIP1L* SNP on chromosome 23 that was associated with ACL rupture. Additionally, there was one SNP on chromosome 24 that was associated with both color and ACL rupture that was within a noncoding region. Functional annotation clustering with DAVID did not identify significant pathway enrichment.

**Table 3:** SNPs associated with the multivariate phenotype of ACL rupture and coat color in the Labrador Retriever using Bayesian GWAS with mvBIMBAM [36]

Chr	Position	Number of SNPs	BF(mult)	BF(ACLR)	BF(color)	PPE(ACLR)	PPE(color)	Interpretation of association	Gene
2	71,101,534–71,129,159	4	3.39–3.76	-0.25-(-0.19)	3.61–4.04	0.41	1.00	color	Non-coding
3	6,904,814	1	3.12	-0.22	3.51	0.49	1.00	color	Non-coding
5	63,697,949	1	31.05	-0.32	31.30	0.34	1.00	color	*TCF25*
5	64,529,952	1	22.09	-0.17	22.55	0.51	1.00	color	*TRAPPC2L*
5	65,740,974	1	21.88	-0.24	22.30	0.46	1.00	color	Non-coding
5	62,868,802	1	20.84	-0.14	20.43	0.29	1.00	color	*SLC25A33*
5	65,726,354	1	20.24	-0.23	20.67	0.47	1.00	color	*FBXO31*
5	64,048,349	2	17.07–17.34	-0.29	17.33–17.59	0.35	1.00	color	*ANKRD11*
5	63,957,203	1	14.39	-0.08	14.86	0.56	1.00	color	*CPNE7*
5	63,097,333	1	12.20	-0.29	12.56	0.41	1.00	color	*CTNNBIP1*
5	63,988,836	1	11.13	-0.20	11.55	0.49	1.00	color	*SPG7*
7	32,703,446	1	4.52	-0.04	4.89	0.57	1.00	color	*RGS7*
10	23,299,673	1	3.04	-0.12	3.26	0.46	1.00	color	*FAM109B*
10	35,494,733	1	3.03	-0.03	3.10	0.51	1.00	color	*SULT1C4*
16	26,472,996–26,477,692	3	3.27–3.91	-0.25-(-0.14)	3.43–4.17	0.40–0.43	1.00	color	*ADAM9*
23	34,304,346–34,308,607	3	3.08–3.11	3.42–3.46	-0.23-(-0.22)	1.00	0.45	ACLR	***DZIP1L***
24	40,095,242	1	3.03	0.81	2.10	0.91	1.00	color and ACLR	Non-coding
26	27,293,677–27,476,357	7	3.14–4.36	-0.25-(-0.04)	3.46–4.61	0.40–0.58	1.00	color	Non-coding
26	27,898,308–27,910,578	3	3.09–3.20	-0.26-(-0.25)	3.43–3.55	0.42–0.44	1.00	color	*BCR*
26	27,762,329	1	3.02	-0.22	3.26	0.42	1.00	color	*RTDR1*

Note: SNPs with significant associations were found on chromosomes 2, 3, 5, 7, 10, 16, 23, 24, and 26. There were 556 significant SNPs on chromosome 5, the top 10 are listed in this table. ACLR, anterior cruciate ligament rupture. Genes within ±25 kb of each SNP are reported

## Discussion

Multivariate GWAS improves power to detect loci with weaker associations with disease risk. Multivariate analysis of ACL rupture case and control Labrador Retrievers of differing coat color revealed associations with several ACL rupture candidate genes after GEMMA and mvBIMBAM analysis, consistent with a polygenic disease. This study validated *ACSF3* and *DZIP1L* [19] as candidate genes, discovered other novel candidate loci, and revealed association with many genes in a chromosome 5 locus.

Considering coat color as a potential risk factor in the development of ACL rupture is a novel approach. Although the exact mechanisms remain unknown, coat color has been found to influence the risk of other disease processes and behavior patterns in the dog [24, 25, 42]. Since ACL rupture is a complex disease, it is possible that coat color genes may have small effects on disease risk, or that other genes that influence risk are inherited together with coat color genes through LD.

The region of the genome that displayed the strongest association was on chromosome 5. There were numerous SNPs that met genome wide significance in both GEMMA and mvBIMBAM analysis. This locus contained many associated genes with proximity to the *MC1R* gene. In both multivariate analyses, the most significant SNP was within the *TCF25* gene. This gene encodes a transcription factor that acts as a transcriptional repressor and is important during embryonic development. It is not currently known to have effects on ACL rupture or coat color, or to directly influence biological pathways that precede disease development. Interestingly, earlier research [43] and our analysis suggests that *TCF25* is in LD with *MC1R*. Mutations in *MC1R* control yellow versus chocolate or black coat color in Labrador Retrievers [21, 22]. *MC1R* is also known to be expressed within articular cartilage [44], and melanocortins promote anti-inflammatory states within joints [26, 28]. Nonfunctional MC1 receptors have been linked to the development of osteoarthritis in mice [29]. Knee osteoarthritis is an inflammatory process that is also associated with ACL rupture in dogs [4]. LD between *TCF25* and *MC1R* may indicate that the effect of this locus is mediated by *MC1R*. Defective MC1 receptors may promote an inflammatory state within the knee that leads to cruciate ligament fiber rupture. It should be noted that no genome-wide associations were found within the *TYRP1* gene, which also controls coat color in Labrador Retrievers, so it is unlikely that the association near *MC1R* is an unintended consequence of including coat color as an additional phenotype.

There were many other significant SNP associations within the haplotype block on chromosome 5 that were located within or near other candidate ACL rupture genes. Interestingly, one of these genes, *ACSF3*, has been previously associated with ACL rupture [19]. *ACSF3* is differentially expressed in ligament [19] and may influence risk of rheumatoid arthritis [45]. *ANKRD11* is another interesting gene in LD with the most significant SNP. This gene regulates cell proliferation and apoptosis. Maladaptive responses to injury in joints could lead to increased inflammation or structural changes that compromise joint homeostasis. Other genes in LD in this locus, including *DPEP1, LZIC, SLC7A5, PIK3CD, RPL13*, and *TNFRSF25*, have been associated with OA or are involved with processes that could link them to ACL rupture pathogenesis [46–54]. *PIK3CD* and *TNFRSF25* regulate lymphocyte development, which could play a role in ACL rupture since lymphoplasmacytic inflammation is seen within affected knees [55–57]. *CLSTN1* is associated with weight and obesity, which are risk factors for ACL rupture development [58].

In both analyses, significant SNPs were located within multiple non-coding regions or lncRNA sequences. These sequences are thought to have regulatory effects on transcription, translation, or post-translational portions of protein production [59, 60]. Effects of regulatory SNPs in the risk of complex canine diseases are not understood. In complex diseases such as ACL rupture, many small-effect SNPs combine to influence disease risk. It is plausible that regulatory mechanisms play a role in gene expression and ultimately influence disease risk. Gene regulation effects on protein production and cell signaling have been implicated in changes to the extracellular matrix, ligament, and cartilage homeostasis, as well as ACL rupture pathophysiology [61]. Other than those on chromosome 5 and the associations within the uncharacterized or non-coding regions, GEMMA analysis identified several additional ACL rupture candidate genes. On chromosome 23, there was a significant SNP located near the *MSL2* gene. *MSL2* is responsible for histone acetylation and gene activation, as well as cellular responses to damage, such as apoptosis [62], and could modulate inflammatory responses leading to ligament fiber rupture [4, 5].

Due to the different statistical approaches of GEMMA and mvBIMBAM programs, it is not surprising that unique SNPs were discovered in each analysis. mvBIMBAM revealed significant associations with 26 SNPs that were not identified by GEMMA, most being associated with coat color. A chromosome 10 locus included the genes *FAM109B* and *SULT1C4*. *FAM109B* plays a role in endocytic trafficking [63]. It is expressed in many tissues including skin melanocytes and keratinocytes [64], which could explain association with coat color. *RGS7* on chromosome 7, *SULT1C4* on chromosome 10, *ADAM9* on chromosome 16, and *RTDR1* and *BCR* on chromosome 26 have no known association with coat color.

*DZIP1L* on chromosome 23 was associated with ACL rupture only, which validates earlier work [19]. *DZIP1L* encodes a protein found in the transition zone of cilia, and mutations have been associated with autosomal recessive polycystic kidney disease, as well as craniofacial deformities and polydactyly [65]. Cilia are found in chondrocytes, fibroblasts, and other connective tissue cells. Primary cilia of connective tissues are within the extracellular matrix and transduce chemical stimuli, mechanical stimuli, or respond to growth factors to control homeostasis, fibroblast migration, and cell cycling [66]. *DZIP1L* mutations resulting in ciliary dysfunction could influence ligament mechanotransduction and cause dysfunction in fibroblast homeostasis.

Through the inclusion of coat color as an additional phenotype, several genes were surprisingly associated with Labrador Retriever coat color. There are two genes that are known to control coat color in Labrador Retrievers [21, 23] as well as many other genes that play a role in coloring patterns, fur length, hair structure, and other related characteristics in dogs [41, 67–69]. Besides these, there have been numerous other loci with effects on coat color in other animal models [70] that have not been studied extensively in dogs. Therefore, it is plausible that some of the coat color gene associations in the current study may influence pathways that determine phenotypic appearance of dogs in ways that are not currently understood. Future research is warranted in this area to determine effects on skin, hair follicles, strands, or related structures that may explain the associations found in this study. There were many unique additional observations in this study. Differences in SNP discovery across studies are likely due to a difference in selection of subject breeds, study design, and analytical approaches [12, 17–19, 71].

In conclusion, GWAS using multivariate linear mixed model and Bayesian model approaches has identified several novel variants associated with ACL rupture and coat color in the Labrador Retriever. The study has also identified two variants, *ACSF3* and *DZIP1L*, that were validated from previous studies [19], suggesting that these genes merit additional investigation. Associated genes in this study have effects on bone and cartilage pathology, inflammatory pathways, metabolism, development, and gene expression and regulation, supporting the complexity of ACL rupture [12]. Although coat color has been linked to many other disease states, this is the first study to examine the relationship between coat color and ACL rupture in the Labrador Retriever. Canine ACL rupture is an important model for human ACL rupture [2], which is also a heritable disease [72] that leads to fatigue injury of ACL fibers and eventual non-contact rupture [2, 73]. Results from this study have translational value for the development of treatment and prevention strategies in both species, particularly polygenic risk score prediction of disease risk in both species.

### Electronic supplementary material

Below is the link to the electronic supplementary material.

Supplementary File S1. SNP GWAS associations with the multivariate phenotype using the GEMMA algorithm [35].

Supplementary File S2. SNP GWAS associations with ACL rupture and coat color phenotypes using the mvBIMBAM algorithm [36].

## Data Availability

The SNP data set used for this analysis cannot be fully shared publicly because of restrictions relating to development of a commercial genetic screening test for ACL rupture in the dog at the University of Wisconsin-Madison. ACL rupture phenotypes are proprietary. The datasets generated and analyzed in this study are available in the Dryad repository at 10.5061/dryad.brv15dvfw.
